# Rex Retroelements and Teleost Genomes: An Overview

**DOI:** 10.3390/ijms19113653

**Published:** 2018-11-20

**Authors:** Federica Carducci, Marco Barucca, Adriana Canapa, Maria Assunta Biscotti

**Affiliations:** Dipartimento di Scienze della Vita e dell’Ambiente, Università Politecnica delle Marche, 60131 Ancona, Italy; f.carducci@univpm.it (F.C.); m.barucca@univpm.it (M.B.); a.canapa@univpm.it (A.C.)

**Keywords:** repetitive DNA, transposable elements, retroelements, rex element, teleost

## Abstract

Repetitive DNA is an intriguing portion of the genome still not completely discovered and shows a high variability in terms of sequence, genomic organization, and evolutionary mode. On the basis of the genomic organization, it includes satellite DNAs, which are organized as long arrays of head-to-tail linked repeats, and transposable elements, which are dispersed throughout the genome. These repeated elements represent a considerable fraction of vertebrate genomes contributing significantly in species evolution. In this review, we focus our attention on Rex1, Rex3 and Rex6, three elements specific of teleost genomes. We report an overview of data available on these retroelements highlighting their significative impact in chromatin and heterochromatin organization, in the differentiation of sex chromosomes, in the formation of supernumerary chromosomes, and in karyotype evolution in teleosts.

## 1. Repetitive DNA

**Figure 1:**
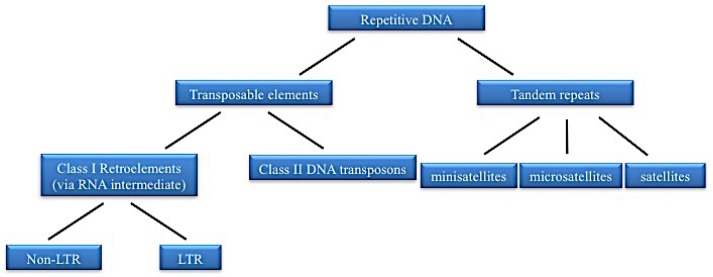
Major divisions of repetitive DNA sequences in the eukaryotic genome. Transposable elements are widespread in the genome and are classified in Class I or Retroelements that require an RNA intermediated into the transposition mechanism and Class II or DNA transposons that move via DNA. Tandem repeats are made up of sequences organized in long array and, based on monomer size, minisatellites, microsatellites, and satellites are distinguishable.

Repetitive DNA is made up of sequence motifs repeated hundreds or thousands of times in the genome and constitutes the major proportion of all the nuclear DNA in most eukaryotic genomes representing in most species more than half of the total DNA content in the cell nucleus [1,2]. It includes satellite DNAs (satDNAs), which are organized as long arrays of head-to-tail linked repeats, and transposable elements (TEs), which are dispersed throughout the genome [3,4,5,6] (Figure 1).

SatDNAs are localized at telomeric, centromeric or pericentromeric regions. Little is known about their possible involvement in biological or functional processes. However, several structural and functional roles have been proposed [7] even if their role is not completely understood. The localization at the centromere suggests potential involvement in centromeric DNA packaging [8,9], chromosome segregation during mitosis and meiosis, pairing of homologous chromosomes, sister chromatid attachment, and kinetochore formation [10]. Recently, a growing body of evidence suggests that satDNA transcripts play a role in heterochromatin formation and maintenance at both centromere and telomere, with a direct impact on karyotype evolution [7]. Comparative studies on the nature and localization of satDNAs between species have revealed that some satDNAs are extremely conserved [11,12,13] while others show a wide range of variability [14,15].

TEs are genetic elements capable of proliferating and inserting themselves into novel locations of the host genome. They can be divided into autonomous or non-autonomous [4]. In the first case, they contain functional sequence coding for the proteins required for their propagation while, in the second case, they use enzymes synthetized by other transposable elements. TEs are also classified as retrotransposons (Class I) or DNA transposons (Class II) on the basis of their transposition mechanism. In particular, retrotransposons are characterized by an RNA intermediate that is then reverse transcribed into complementary DNA, using a copy and paste mechanism. This category includes the long terminal repeat (LTR)-containing retroelements and non-LTR containing retroelements. The first report of LTR was in avian sarcoma virus by Shine et al. (1977) [16]. LTRs are constituted by identical sequences of hundreds of thousands of nucleobases repeated in tandem. In LTR retrotransposons, the promoter is located within 5′-LTR and recruits a RNA polymerase. In this case, the transposition requires the complete synthesis of all Reverse Transcriptase-machinery components constituted by *gag* protein, reverse transcriptase (RT), protease, RNAse H, and integrase and produced as a poly-domain “precursor protein”. After the RT-mediated cDNA synthesis, integrase inserts the cDNA into a new position of the genome. On the other hand, non-LTR retroelements are mainly represented by Long Interspersed Nuclear Elements (LINEs) and Short Interspersed Nuclear Elements (SINEs). LINEs are non-LTR retrotransposon that may contain one or two Open Reading Frames (ORFs) according to Wicker et al. (2007) [4]. One of these two ORFs encodes for an endonuclease (EN) that is the mediator of the transposition. SINEs are retrotransposed elements that originated by the reverse transcription of Pol III transcripts [1] and do not encode for proteins, thus, for their retrotransposition, they need LINE reverse transcriptase [17]. DNA transposons include: subclass I in which both DNA strands are cleaved (Terminal inverted repeat transposons and Crypton elements) and transposition follows the canonical cut and paste mechanism; different is the case of the subclass II including Helitrons and Maverick/Polinton elements in which the transposition follows the copy and paste mechanism [4,5]. Among non-autonomous transposons miniature inverted transposable elements (MITEs) exploit transposase encoded by autonomous elements to transpose [1].

It is currently known that TEs represent a considerable fraction of the genome [18] and have had a significant influence on the evolution of genomes, through their involvement in chromosome rearrangements and sexual chromosome differentiation [19,20]. TEs can play a role in the reorganization of the genome being co-opted or exapted to form new exons and regulatory sequences and even new RNA and protein-coding genes [21,22,23,24]. Moreover, TEs show lineage-specific diversity in terms of composition, content and age, contributing to genome plasticity and the evolution of the host genome [25]. They are also able to spread between reproductively isolated species through horizontal transfer mediated by viruses, sensibly increasing the complexity of transposon evolution on genomes [26,27].

There is much evidence that satDNAs and TEs are interconnected with each other. Indeed, several papers report the sharing of sequence similarity between satDNAs and TEs, suggesting a complex mutual relationships influencing the genome architecture [28,29,30].

TEs exhibiting a great variety in structure, size and mechanisms of transposition can represent powerful drivers of species diversity [31,32]. Most teleost genomes are predominantly composed of DNA transposons, exception made for tetraodon and stickleback, in which no prevalence of particular type of TEs has been evidenced and for fugu which is rich in LINEs and SINEs. Although most TE superfamilies have been reported in teleost genomes, many DNA transposons are absent in some species, in particular fugu, tetraodon, stickleback, tilapia, and platyfish. Interestingly, some teleost genomes (zebrafish, tilapia, stickleback, and pufferfish) are particularly rich in recent TE copies while others rich in ancient copies. This difference has been related to the time in which bursts of transposition occurred during evolutionary history of species [33].

In this review, we report an overview of data available on Rex elements, a lineage specific retroelement, and highlight their significative impact on the evolution of teleost karyotype.

## 2. Rex Elements in Teleosts: Importance and Structure

**Figure 2:**
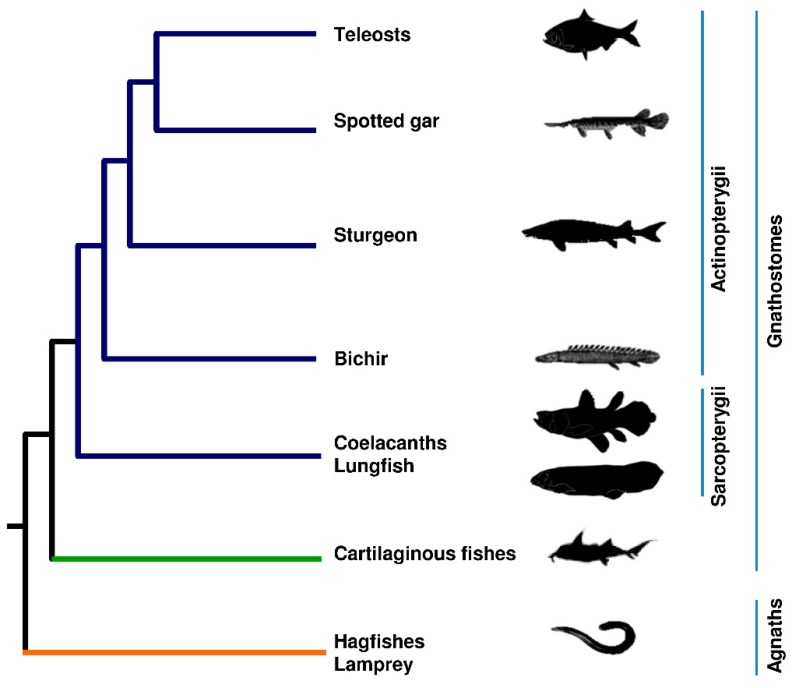
Evolutionary relationships between jawless fishes, cartilaginous fishes, basal sarcopterygian fishes, and actinopterygians. Orange branch represents jawless fishes; green branch represents Chondrichthyes; blue branches represent Osteichthyes.

Jawless fishes (hagfishes and lampreys), cartilaginous fishes (sharks and rays), and bony fishes (coelacanth, lungfishes and ray finned fishes) are highly diverse animals that have adapted successfully to a wide range of environments. Among them, teleosts represent more than 99.8% of ray-finned fishes which include also bichirs and sturgeons (Figure 2 and Figure S1). The genome of these organisms evolves extremely rapidly, suggesting the presence in their genomes of very powerful evolutionary tools. Indeed, they possess a rich repertoire of transposable elements, with highly diverse content between lineages and even between species [25,34]. The study of the organization and evolution of fish genomes is attractive to assess the impact of mobile elements on vertebrate genomes and the evolutionary mechanisms possibly underlying biodiversity in this taxon.

**Figure 3:**
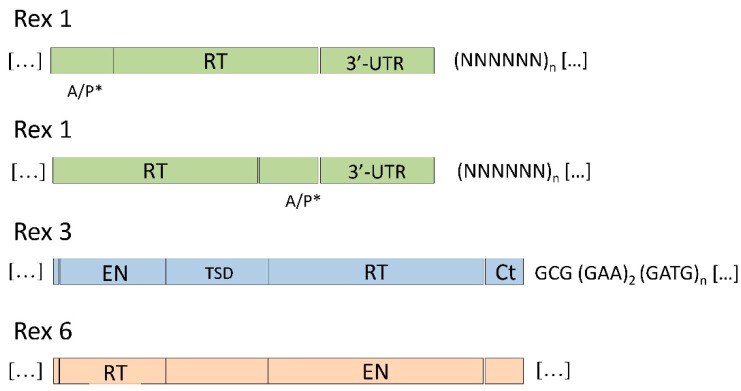
Main features of the Rex1 retroelement (A/P*, apurinic/apyrimidinic site that can be located upstream or downstream the RT-encoding region; RT, reverse transcriptase-encoding region; 3′UTR), Rex3 (EN, endonuclease-encoding region; TSD, target site duplication; RT, reverse transcriptase-encoding region; Ct, C-terminal domain–encoding region; tail consisting of tandem repeats of the sequence GCG (GAA)2 (GATG)n, where *n* = 8–17 [35], and Rex6 (RT, reverse transcriptase-encoding region; EN, endonuclease-encoding region).

Rex1, Rex3, and Rex6, first isolated in the fish model *Xiphophorus* (Figure 3), are non-LTR retrotransposons widely distributed among teleost genomes and were active during the evolution of this lineage [26,27,35]. Although these three retrotransposons are usually analyzed together, no evidence has been reported about their evolutionary relationship.

The first Rex element identified was Rex3. Using a PCR-mediated amplification starting from a fragment of the Rex3, many copies of this retroelement were evidenced by Volff and co-workers [35]: Rex3a-XmJ, Rex3b-XmJ, and Rex3c-XmJ (Table 1). The comparison of Rex3 retroelement given in this work [27,35] for the platyfish genus *Xiphophorus* with that of *Fugu rubripes* and *Oryzias latipes* allowed us to delineate a more general profile of this mobile element. Standard features for the Rex3 retroelement are the presence of Reverse Transcriptase (RT) domains, no LTR flanking regions, a 3′-end of Rex3 consists of two GAA repeats followed by GATC tandem repeats, and the stop codon that terminates the C-terminal-domain-encoding sequence is located at a distance of only 3 nt (for the *Xiphophorus* 5′-GCG-3′) upstream of the first GAA repeat (Figure 3).

In particular, Volff and colleagues [35] underlined that the GATC tail is the *signature* for the Rex3 element, different from those reported for the RTE-like elements.

Always considering as starting point an insert sequence derived from the Y chromosome of *X. maculatus*, Volff and colleagues [26] identified a sequence of a truncated copy of a non-LTR retrotransposon that they called Rex1-XimJ (Retroelement of the *X. maculatus*, Rio Jamapa). Rex1-XimJ is closely related to the *CR1* clade of the LINE elements and to *Babar* elements of *Battrachoccottus baikalensis*. An extended research in public sequence databases allowed them to reconstruct three partial sequences of Rex1 from the *F. rubripes* genome (Rex1-FurA:C) and one *Rex1* variant in *Tetraodon nigroviridis* (Rex1-Ten).

Main features of the Rex1 element can be derived from the study of Volff and co-workers [26]: apurinic/apyrimidinic (AP) endonuclease-encoding sequences located upstream (or downstream) of the RT-encoding domain (Figure 3), frequently removed from the uncomplete reverse transcription; a conserved 3′-untranslated region followed by an oligonucleotide sequence of variable length. No endonuclease-encoding domain can be detected upstream of the RT-encoding sequence for the *X. maculatus*.

Rex6 is a member of the R4 family specialized for insertion in rRNA genes of its host [59]. This mobile element is a non-LTR retrotransposon and its structure includes a RT and a putative restriction enzyme-like endonuclease (RLE) (Figure 3).

## 3. The Impact of Rex Retroelements in Teleost Genomes: An Overview on Identification, Chromosome Mapping, and Karyoevolution.

The teleost genome presents a wide ability to incorporate transposable elements from 6% in the pufferfish tetraodon to 55% in zebrafish and a higher TE diversity than other vertebrate genomes [33]. This is reflected in the genomic variety and different evolutionary trends of chromosomes. Indeed, they present huge chromosome diversity with interspecific variation in diploid numbers, the presence or absence of sex and supernumerary chromosomes [60]. Several authors have performed cytogenetic analyses to investigate the physical mapping of repeated sequences on the teleost chromosomes and to understand the organization of TEs and their role in the diversification of their genomes.

Volff and co-workers [35] evidenced for the first time in *Xiphophorus maculatus* three new RT-carrying retrotransposons named as Retroelements of *Xiphophorus maculatus* Rex1, Rex2, and Rex3.

Initially, Rex1 was not assigned to a major group of retroelements, whereas Rex3 was detected by the same group in all members of Poecilidae as well in other teleosts including *Fundulus* sp., *Siniperca chuatsi*, *Anguilla anguilla*, *Cyprinus carpio*, and *Danio rerio* and in cichlids from different geographical regions. In the sturgeon *Acipenser sturio*, this mobile element was not found (Table 1). The absence of identification of these elements in early-branching actinopterygians led to assume that the presence of these TEs was confined to teleosts. Volff et al. [35] suggested the active role of Rex3 elements and the possible position of these elements at the base of the origin of SINEs.

Interestingly, a Rex3 phylogenetic analysis was discussed by Volff and colleagues in a work published in 2001 [27] in which the authors concluded that the phylogeny of Rex3 does not follow the accepted fish phylogeny. This was explained by the presence of several different ancient Rex3 lineages in teleost genomes that diverged before their actual host genomes did. The author affirmed that Rex3 lineages are probably not very ancient and that the low rate of synonym substitution found in Rex3 sequences from different species can be related to the horizontal transfer during ray-finned fish genome evolution.

An exhaustive phylogenetic analysis [26] was made for the Rex1 element focusing on putative RT sequences. However, Rex1 was not found in carp, trout, pike and zebrafish suggesting a very complex and dynamic role of retrotransposon on vertebrate genomes. The lack of identification of Rex1 in these species suggested two possible mechanisms to explain the evolution of this element: the first one might be the frequent loss and rapid sequence divergence, while the second one is represented by the possibility of horizontal transfer.

After these first reports on Rex elements, other groups started to identify and physically map these retroelements in many teleosts to get new insights onto the localization of these mobile elements in teleost chromosomes.

Fluorescent in Situ Hybridization (FISH) analyses of *Tetraodon nigroviridis* genome using Rex3 as probe evidenced multiple and intense signals mostly in short arms and pericentromeric regions of small submeta- or subtelocentric pairs [56,57]. Moreover, thin hybridization spots were present throughout the chromosomes suggesting the presence of multiple interstitial copies in euchromatic regions [57]. In the compact genome of this teleost, Rex1 elements were also identified in the heterochromatin in the framework of a study conducted to investigate the global and local organization of repeat sequences [58].

In 2004, Ozouf-Costaz and co-workers [51] published a work in which authors reported Rex retroelements identified in several species of Antarctic fish belonging to five families of the suborder of the Nothotenioidei. They mapped Rex1 and Rex3, evidencing an abundance for this latter, which is also homogeneously spread all over the chromosomes. Interestingly, *Notothenia coriiceps* represents an exception among the investigated species since it showed a higher peri-centromeric compartmentalization of Rex retroelements. These findings suggest a role of transposons in the molecular differentiation of karyotype.

Cioffi and colleagues [42] performed molecular cytogenetic analyses to get insights into the karyoevolution in *Erythrinus erythrinus*, a species having a karyotypic diversity among populations, with four currently identified karyomorphs (A–D). Samples from allopatric populations of karyomorphs A and D showed differences in the distribution of Rex3 and 5S rDNA. In karyomorph A, Rex3 signals were detected in the centromeric regions of only two acrocentric chromosomes, while, in karyomorph B, they were found in 22 acrocentric chromosomes in females and 21 in males. Moreover, Rex3 and 5S rDNA co-localized in the centromeric region of several acrocentric chromosomes and at the centromeric position of the large metacentric Y chromosome in the karyomorph D. This result together with the localization of the telomeric (TTAGGG)n repeats at centromeric region suggested that the Y chromosome derived by centric fusion of acrocentric pairs.

Rex3 element was also hybridized onto the chromosomes obtained from two Brazilian allopatric populations of *Astyanax bockmanni* showing 2*n* = 50 chromosomes but diversities in fundamental number [38]. The double-FISH of Rex3 with 18S rDNA evidenced a co-localization of these elements mainly at telomeric heterochromatic regions. However, conspicuous differences among the representatives of the two populations were evidenced. Indeed, 18S rDNA and Rex3 syntenic blocks were detected in pairs 3 and 6 in individuals from Água da Madalena and not in individuals from Capivara River. Individuals from this area showed 18S rDNA and Rex3 syntenic blocks in pair 21, while only Rex3 sequences were present in the correspondent pair of representatives of Água da Madalena. The co-localization of Rex3 and 18S rDNA was explained by suggesting that Rex3 retrotransposons contribute to disperse rDNA or simply Rex3 elements follow the spread of NOR.

The physical mapping of Rex elements performed in six natural population of the neotropical fish *A. bockmanni* evidenced different patterns suggesting that different mechanisms drive the spread of repetitive sequences among the analyzed population [39]. Rex1 and Rex6 showed small clusters scattered on most chromosomes of the six populations. Rex3 exhibited a different distribution pattern among the populations being mainly located in one acrocentric chromosome pair of two populations and in large bocks in more than one chromosome pair in the other populations. This element was also mapped in the heterochromatic blocks of the congeneric species *A. fasciatus* sampled from the Tietê river [40]. Interestingly Rex1, Rex3, and Rex6 signals were detected in the acrocentric B chromosome of *A. bockmanni* specimens sampled from the Alambari river [39]. In addition, Fantinatti and colleagues [43] reported the detection of Rex1 and Rex3 signals in the B chromosomes of *Astatotilapia latifasciata*. The presence of Rex transposons in the supernumerary chromosome of these species could suggest their involvement in the origin and possibly in maintenance of the additional chromosome. Indeed, the origin of B chromosomes has been related to the accumulation of repeated DNA sequences essential to achieve a minimum size required to be considered a functional chromosome [61].

The co-localization of Rex retrotransposons with rDNA elements was also evidenced in three sympatric species of the genus *Hypostomus* belonging to the family Loricariidae after co-hybridization between Rex1 and 5S rDNA sequences [55]. However, the not appreciable accumulation of Rex1 in the heterochromatin of most chromosomes suggested that they do not play a key role in the structure and organization of heterochromatic area as observed in cichlids [44].

Furthermore, in the family Loricariidae analysing *Hisonotus leucofrenatus*, *Pseudotocinclus tietensis,* and *Parotocinclus maculicauda* the chromosome mapping of Rex1 and Rex3 elements evidenced dispersed signals on all chromosomes not only in heterochromatin but also in euchromatin [54] contradicting the general notion according to which retrotransposon elements are preferentially accumulated in the heterochromatic regions in teleosts.

The heterochromatic and euchromatic localization of Rex elements was also reported by Costa and colleagues [36] which investigated by fluorescence in situ hybridization the chromosome distribution of Rex1 and Rex3 in *Rachycentron canadum*, a species of commercial interest living in tropical seas and largely employed in marine fish farming. Their findings revealed that Rex1 and Rex3 are preferentially associated with telomeric heterochromatin, and Rex3 shows an additional distribution in euchromatic regions of the chromosomes.

Recently, Favarato et al. [53] reported a similar distribution pattern also in seven species of the bristlenose catfish (genus *Ancistrus*). Indeed, in this work, the authors made an exhaustive physical mapping of Rex1, Rex3, and Rex6, evidencing a cluster distribution for each of the three retroelements. Sequences of Rex1 were detected in pericentromeric regions of chromosomes as well as for the Rex3 element, which in addition showed conspicuous markings on the terminal regions of chromosomes. Rex6 was preferentially located at terminal chromosomal regions, except for the *Ancistrus* sp. “purus” in which the localization is mainly pericentromeric. The preferential heterochromatic localization was related by Favarato et al. [53] to the epigenetic mechanism at the base of regulation of these retroelements that acts to avoid an excessive propagation of the same in the genome. Focusing attention to this point, the localization of Rex elements also in euchromatic regions in members of the *Ancistrus* genus may suggest a major importance in the genomic evolution of these teleost species. The transposition of TEs in euchromatic regions can provoke mutations, variation in gene expression and DNA recombination affecting the organization of genomic architecture [62,63,64].

The chromosome localization of Rex1 was also investigated in nine Bagridae species from Thailand: Hemibagrus filamentus, H. nemurus, H. wyckii, H. wyckioides, Mystus atrifasciatus, M. multiradiatus, M. mysticetus, M. bocourti, and Pseudomystus siamensis [52]. The retrotransposon element Rex1 showed a scattered distribution pattern throughout many chromosomes, but in M. atrifasciatus, M. multiradiatus and Hemibagrus spp., besides dispersed signals in heterochromatic and euchromatic regions, an unexpected accumulation was detected in some chromosome pairs [65].

It is known that exists a close relationship between heterochromatin formation and sex chromosome differentiation. Indeed, sex chromosomes are characterized by the absence of recombination that results in an accumulation of repetitive sequences on one of the pair of sex chromosomes [65]. In this context, 12 repetitive DNA sequences were mapped to get insights into the differentiation of the Z and W chromosomes in the species *Triportheus trifurcatus* [41]. Among the repetitive DNA sequences analyzed, Rex1, Rex3, and Rex6 showed a dispersed distribution on most chromosomes including Z chromosome in *T. trifurcatus*. Moreover, Rex3 signals were detected throughout the whole W chromosome and in particular at the telomeric region of the long arm; Rex1 showed more intense signals in the long arms of the W chromosome; and Rex6 was restricted to a few cluster in short arms of the W chromosome.

Interstitial signals of Rex1 and Rex3 were found also in the W chromosomes of *Leporinus elongates*, *L. macrocephalus*, and *L. obtusidens*, three species of the family Anostomidae [37] and Rex3 signals were detected in the long arm of Y chromosome in *Chionodraco hamatus* [51] suggesting a possible involvement of these retrotransposons in sexual differentiation [37,51].

Among teleosts, the family Cichlidae comprises between 2000 and 3000 species that inhabit rivers and lakes in tropical and subtropical regions of Africa and the Americas, as well as India and Sri Lanka. This group of teleosts represents an excellent evolutionary model due to its adaptive radiation and its ecological and behavioral diversity. The investigation of TEs in the genomes of cichlids could strongly contribute to the understanding of the basal evolutionary mechanisms involved in the generation of phenotypic and genetic variability of these organisms.

Teixeira and colleagues [48] investigated the organization of five repeated DNA elements in the genome of the cichlid *Cichla kelberi*. Sequences belonging to Rex1, Rex3, and Rex6 were isolated in *C. kelberi* and mainly localized in the centromeric heterochromatin. Rex1 and Rex3 shared a similar distribution pattern being localized both at centromere and spread throughout the long euchromatic arms of the first and third chromosome pairs. Rex6 was not only present at centromere but also dispersed along the chromosomes of *C. kelberi*.

FISH analyses using Rex3 retrotransposon as probe in mitotic and meiotic chromosomes of three species of the genus *Symphysodon* were performed by Gross and colleagues [50] to investigate the role of repetitive sequences in the generation of phenotypic and genetic variability typical of wild Discus populations. The authors reported a co-localization of Rex3 signals with heterochromatin in *S. aequifasciatus*, *S. discus,* and *S. haraldi* suggesting a role of TEs in karyotype differentiation in the *Symphysodon* genus.

Rex1, Rex3, and Rex6 sequences were mapped onto the chromosomes of four African (Oreochromis niloticus, Haplochromis obliquidens, Hemichromis bimaculatus, and Melanochromis auratus) and four South American (Astronotus ocellatus, Chaetobranchus flavescens, Satanoperca jurupari, and Heros efasciatus) cichlid species [44]. The cytogenetic analyses evidenced that Rex elements were mainly compartmentalized in the pericentromeric heterochromatin of most chromosomes in all cichlid species investigated. Interestingly in the Nile tilapia Oreochromis niloticus, Rex retroelements were concentrated in the largest chromosome pair recognized as sex chromosome [49] and probably derived by fusions. This finding underlines the role of transposable elements in chromosome rearrangements and also in sex chromosomes differentiation [37,41,51]. Dispersed signals of the analyzed retroelements were also detected in euchromatic regions of several chromosomes, except for C. kelberi showing an accumulation of Rex1 and Rex3 in euchromatic regions of only two chromosome pairs, as also evidenced by Teixeira and co-workers [48]. This may be due to its close relation to the ancestral karyotype, given its phylogenetic position.

Overall, the three Rex elements hybridized showed differences in signal intensity that are related to their copy number in the genomes of analyzed species. Independent mechanisms of amplification and removal could have acted after the split of the ancestral cichlid lineages and have been responsible for the differences in the copy number and chromosomal distribution observed in cichlids.

*C. kelberi* was also analyzed by de Freitas Mourão and colleagues [47] together with the congeneric species *C. piquitii,* evidencing a spread distribution of Rex elements on the majority of chromosomes in contrast with findings reported by Teixeira et al. [48] and Valente et al. [44].

Schneider and colleagues [45] coupled physical chromosome mapping of the non-LTR retrotransposons Rex1, Rex3, and Rex6 in five Amazonian cichlid species with the evaluation of the Rex sequence genetic diversity. In addition, in this paper, a scattered localization of Rex1 elements throughout the chromosomes of *Cichla monoculus*, *Astronotus ocellatus*, *Geophagus proximus*, *Pterophyllum scalare*, and *Symphysodon discus* was observed. Moreover, in *A. ocellatus* and *G. proximus*, Rex1 was predominantly distributed in few specific chromosome pairs while for *S. discus* in most of the chromosomes pairs. Overall, conspicuous clusters were present in the terminal and/or centromeric regions in all species analyzed. Rex3 and Rex6 showed intense sites in terminal regions of most chromosomes as well as in centromeric, pericentromeric and interstitial regions. In particular, the localization of Rex3 at the centromeric heterochromatin in *A. ocellatus* was also previously reported by Mazzuchelli and Martins [46] and Valente et al. [44]. In addition, the FISH mapping revealed an association between Rex1, Rex3, and Rex6 and the 18S and 5S ribosomal sites in all the analyzed species, indicating that TEs may have had a role in dispersing rDNAs generating multiple clusters [45]. The sequence analysis of 49 Rex1 clones, 126 Rex3 clones, and 156 Rex6 clones evidenced a more conservation in Rex1 and Rex3 of basal species than the derived species, whereas Rex6 exhibited high substitution rates in both basal and derived species. Moreover, the Bayesian analysis performed for all three Rex elements showed no congruence with the phylogenetic hypothesis described for the group suggesting that TE sequences evolve independently in the genome of their hosts probably as consequence of horizontal transfer [26,27]. In addition, the Rex1 sequences of some teleost species showed high level of similarity, indicating a recent activity of this element. Several copies of Rex3 sequences presented stop codons, suggesting that these elements are inactive.

## 4. Conclusions

Repetitive DNA, given its high variability in terms of sequence, genomic organization, and evolutionary mode, is an intriguing portion of the genome still not completely known [1,2]. In particular, mobile elements represent a considerable fraction of vertebrate genomes contributing significantly in species evolution. Indeed, although these repeated elements can be deleterious for the host due to their spreading mechanisms, they are an important source to generate new exons and regulatory sequences. Between vertebrates, teleost genomes show a higher level of TE diversity than other lineages representing an important evolutionary tool. In this context, the investigation of Rex elements is extremely interesting even more so if we consider that these non-LTR elements are teleost-specific. Overall, the cytogenetic data obtained to date have evidenced that Rex elements are preferentially localized in heterochromatin in particular at telomeric [36,38,41,53], pericentromeric and centromeric regions [42,44,48,51,53] but also in supernumerary chromosomes [39,43]. However, several papers have reported even a surprising euchromatic localization of Rex retroelements [36,48,52,54,57]. This could be related to higher rate of gene-linkage disruption and chromosomal rearrangements more in teleost genomes than mammals [42].

Moreover, regarding chromosomal arrangements, Rex retroelements can be widely spread among chromosomes [39,40,41,45,47,51,52,54] or preferentially located in specific chromosomal regions and/or chromosome pairs [26,36,38,39,40,42,44,45,48,49,51].

However, data here summarized even if restricted to few orders allow to trace a general trend of Rex chromosomal organization for Characiformes, Cichliformes, Perciformes, and Siluriformes, the four teleost orders most studied in the last two decades. Within Characiformes, in species belonging to Anastomidae and Characidae families, for Rex1 and Rex3 elements, a preferential location at telomeric regions can be inferred [37,38,41], while, for the only analyzed species belonging to Erythrinidae family, a centromeric localization has been evidenced [42]. Concerning Cichliformes, in the 18 species investigated, Rex retroelements showed a wide variability in the hybridization signal distribution. Indeed, three species exhibited a spread distribution of Rex retroelements [43,47], four a compartmentalization in specific chromosome pairs [43,45,48], and eight a pericentromeric heterochromatic localization of most chromosomes [44,49]. Focusing on Nototheniidae family belonging to Perciformes, Rex1 and Rex3 signals were spread all over the chromosomes, except for *N. coriiceps*, in which a pericentromeric compartmentalization of Rex retroelements has been reported [51]. Species belonging to Siluriformes showed a very interesting patterns of Rex elements distribution. Most show a scattered profile throughout many chromosomes [40,52,54], while, in all eight species analyzed from the *Ancistrus* genus, Rex3 and Rex6 were preferentially localized at terminal chromosomal regions [53].

Some papers evidenced also the involvement of Rex elements in the differentiation of sex chromosomes [37,41,42,51]. Among the three Rex elements, Rex6, given its different distribution pattern, seems to have played a predominant role in the evolutionary dynamics of sex chromosomes [41].

Although the Rex retroelement information is fragmentary, this review has nevertheless allowed outlining the importance that they have had in determining the genome structure and karyotype evolution in teleosts. In the future, the increasing of information regarding not only cytogenetics, but also the sequence variability in different orders will allow improving the comprehension of the evolutionary history of these intriguing retroelements in teleosts.

## Figures and Tables

**Table 1:** Summarizing scheme of Rex elements in teleost species.

Order, Family and Species	Transposable Element	Reference
**Anguilliformes**		
**Anguillidae**		
*Anguilla anguilla*	Rex3	[35]
*A. japonica*	Rex3	[35]
**Beloniformes**		
**Adrianichthyidae**		
*Oryzias latipes*	Rex1, Rex3	[26,27]
**Carangiformes**		
**Rachycentridae**		
*Rachycentron canadum*	Rex1, Rex3	[36]
**Centrarchiformes**		
**Sinipercidae**		
*Siniperca chuatsi*	Rex3	[35]
**Characiformes**		
**Anostomidae**		
*Leporinus elongatus*	Rex1, Rex3	[37]
*L. friderici*	Rex1, Rex3	[37]
*L. lacustris*	Rex1, Rex3	[37]
*L. obtusidens*	Rex1, Rex3	[37]
*L. macrocephalus*	Rex1, Rex3	[37]
*L. striatus*	Rex1, Rex3	[37]
**Characidae**		
*Astyanax bockmanni*	Rex3	[38]
*A. bockmanni*	Rex1, Rex3, Rex6	[39]
*A. fasciatus*	Rex3	[40]
*Triportheus trifurcatus*	Rex1, Rex3, Rex6	[41]
**Erythrinidae**		
*Erythrinus erythrinus*	Rex3	[42]
**Cichliformes**		
**Cichlidae**		
*Astatotilapia latifasciata*	Rex1, Rex3, Rex6	[43]
*A. ocellatus*	Rex1, Rex3, Rex6	[44]
*A. ocellatus*	Rex1, Rex3, Rex6	[45]
*A. ocellatus*	Rex3, Rex6	[46]
*Chaetobranchus flavescens*	Rex1, Rex3, Rex6	[44]
*Cichla monoculus*	Rex1, Rex3, Rex6	[45]
*C. piquiti*	Rex3, Rex6	[47]
*C. kelberi*	Rex1, Rex3, Rex6	[48]
*C. kelberi*	Rex3, Rex6	[47]
*Cichlasoma labridens*	Rex1	[26]
*Geophagus proximus*	Rex1, Rex3, Rex6	[45]
*Haplochromis obliquidens*	Rex1, Rex3, Rex6	[44]
*Hemichromis bimaculatus*	Rex1, Rex3, Rex6	[26,44]
*Heros efasciatus*	Rex1, Rex3, Rex6	[44]
*Melanochromis auratus*	Rex1, Rex3, Rex6	[44]
*Oreochromis niloticus*	Rex1, Rex3, Rex6	[26,44,49]
*Pterophyllum scalare*	Rex1, Rex3, Rex6	[45]
*Satanoperca jurupari*	Rex1, Rex3, Rex6	[44]
*Symphysodon aequifascistus*	Rex3	[50]
*S. discus*	Rex3	[50]
*S. discus*	Rex1, Rex3, Rex6	[45]
*S. haraldi*	Rex3	[50]
**Cypriniformes**		
**Cyprinidae**		
*Cyprinus carpio*	Rex3	[35]
*Danio rerio*	Rex3	[35]
**Cyprinodontiformes**		
**Fundulidae**		
*Fundulus sp.*	Rex1	[26]
**Poecilidae**		
*Gambusia affinis*	Rex1	[26]
*Girardinus falcatus*	Rex1	[26]
*G. metallicus*	Rex1	[26]
*Heterandria bimaculata*	Rex1	[26]
*H. formosa*	Rex1	[26]
*Phallichthys amates*	Rex1	[26]
*Poecilia formosa*	Rex1	[26]
*Poeciliopsis gracilis*	Rex1	[26]
*Poecilia latipinna*	Rex1	[26]
*P. mexicana*	Rex1	[26]
*Xiphophorus helleri*	Rex1, Rex3	[26,35]
*X. maculatus*	Rex1	[26]
*X. montezumae*	Rex1	[26]
*X. nezahualcoyotl*	Rex1	[26]
**Esociformes**		
**Esocidae**		
*Esox lucius*	Rex1	[26]
**Perciformes**		
**Artedidraconidae**		
*Artedidraco shackletoni*	Rex3	[51]
**Bathydraconidae**		
*Gymnodraco acuticeps*	Rex1, Rex3	[51]
*G. victori*	Rex1, Rex3	[51]
**Bovichtidae**		
*Bovichtus angustifrons*	Rex1, Rex3	[51]
**Channichthyidae**		
*Chionodraco hamatus*	Rex1, Rex3	[51]
*Neopagetopsis ionah*	Rex1, Rex3	[51]
**Cottidae**		
*Battrachocottus baikalensis*	Rex1	[26]
**Nototheniidae**		
*Dissostichus mawsoni*	Rex1, Rex3	[51]
*Notothenia coriiceps*	Rex1, Rex3	[51]
*Patagonotothen tessellata*	Rex1, Rex3	[51]
*Trematomus newnesi*	Rex1, Rex3	[51]
*T. hansoni*	Rex1, Rex3	[51]
*T. bernacchii*	Rex1, Rex3	[51]
*T. pennellii*	Rex1, Rex3	[51]
**Salmoniformes**		
**Salmoniformes**		
*Oncorhynchus mykiss*	Rex1	[26]
**Siluriformes**		
**Bagridae**		
*Hemibagrus filamentus*	Rex1	[52]
*H. nemurus*	Rex1	[52]
*H. wyckii*	Rex1	[52]
*H. wyckioides*	Rex1	[52]
*Mystus atrifasciatus*	Rex1	[52]
*M. multiradiatus*	Rex1	[52]
*M. mysticetus*	Rex1	[52]
*M. bocourti*	Rex1	[52]
*Pseudomystus siamensis*	Rex1	[52]
**Loricariidae**		
*Ancistrus sp. 1 “Purus”*	Rex1, Rex3, Rex6	[53]
*A. sp. 2 “Catalão”*	Rex1, Rex3, Rex6	[53]
*A. dolichopterus*	Rex1, Rex3, Rex6	[53]
*A. aff. dolichopterus*	Rex1, Rex3, Rex6	[53]
*A. dubius*	Rex1, Rex3, Rex6	[53]
*A. maximus*	Rex1, Rex3, Rex6	[53]
*A. ranunculus*	Rex1, Rex3, Rex6	[53]
*Hisonotus leucofrenatus*	Rex1, Rex3	[54]
*Paratocinclus maculicauda*	Rex1, Rex3	[54]
*Pseudotocinclus tietensis*	Rex1, Rex3	[54]
*Hypostomus ancistroides*	Rex1	[55]
*H. strigaticeps*	Rex1	[55]
*H. nigromaculatus*	Rex1	[55]
**Tetraodontiformes**		
**Tetraodontidae**		
*Takifugu rubripes*	Rex1, Rex3	[26,27]
*Tetraodon nigroviridis*	Rex1, Rex3	[26,56,57,58]

## Supplementary Materials

Supplementary Materials can be found at http://www.mdpi.com/1422-0067/19/11/3653/s1. Figure S1: Cladogram showing the relationships of bony fishes [66].
