# Supplementary material for: Rex Retroelements and Teleost Genomes: An Overview
Source: Int J Mol Sci. 2018 Nov 20;19(11):3653. doi: 10.3390/ijms19113653 (PMC6274825; doi:10.3390/ijms19113653)
Supplement: Supplementary file 1 [file ijms-19-03653-s001.pdf]

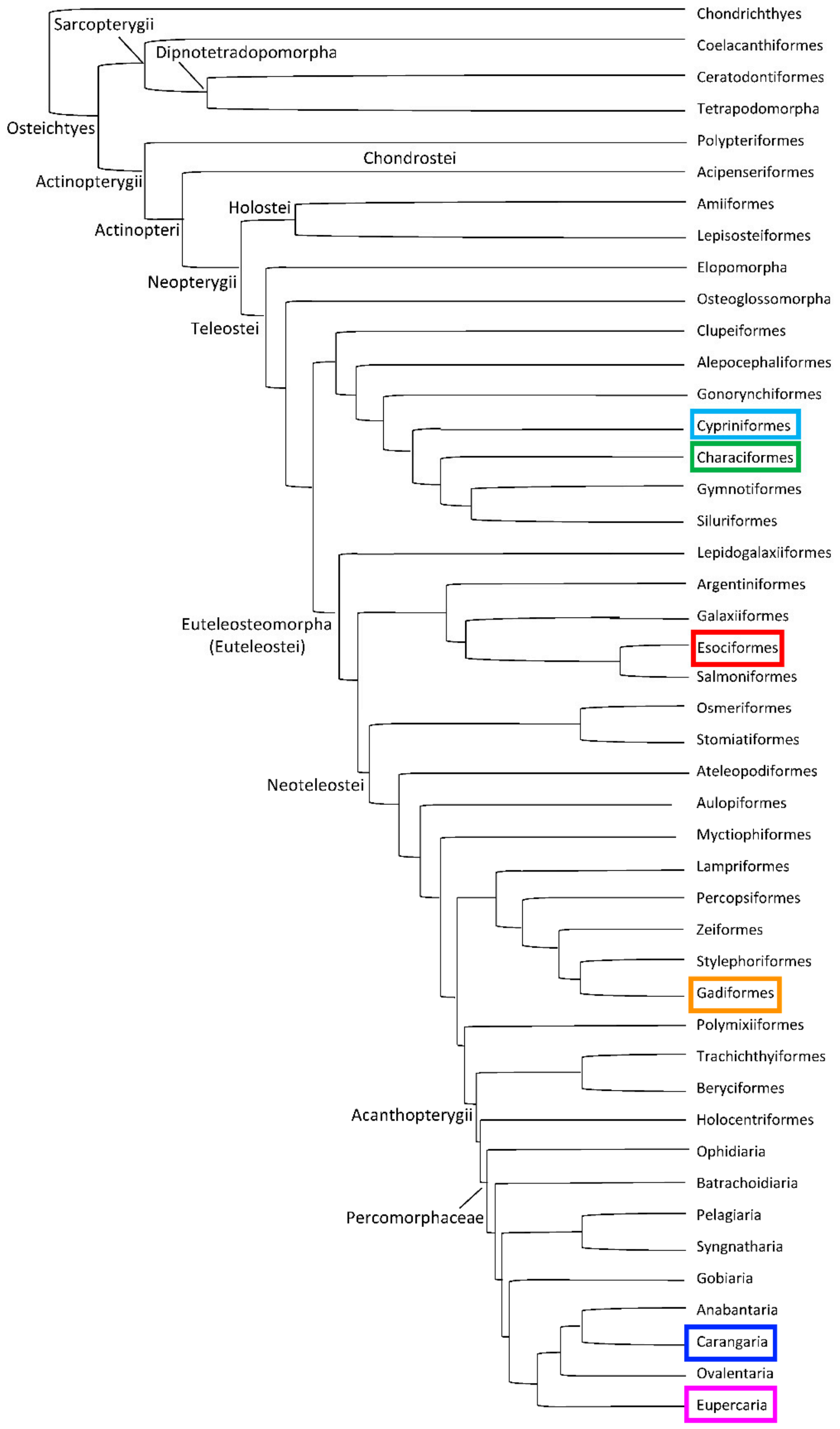

Figure S1. Cladogram showing the relationships of bony fishes [66]. Taxa of species named in the text are highlighted by colored boxes.
